# C-type natriuretic peptide attenuates renal osteodystrophy through inhibition of FGF-23/MAPK signaling

**DOI:** 10.1038/s12276-019-0265-8

**Published:** 2019-07-01

**Authors:** Dong Dong Zhang, Yang Fang Wu, Wei Xia Chen, Yao Xu, Si Yan Liu, Huang Huang Luo, Guang Mei Jiang, Yue Wu, Peng Hu

**Affiliations:** Department of Pediatrics, the First Affiliated Hospital of Anhui Medical University, No. 218 Ji-Xi Road, 230022 Hefei, China

**Keywords:** Experimental models of disease, Kidney diseases

## Abstract

Renal osteodystrophy (ROD) occurs as early as chronic kidney disease (CKD) stage 2 and seems ubiquitous in almost all pediatric patients with CKD stage 5. Fibroblast growth factor (FGF)-23, a bone-derived endocrine regulator of phosphate homeostasis, is overexpressed in CKD and disturbs osteoblast differentiation and matrix mineralization. In contrast, C-type natriuretic peptide (CNP) acts as a potent positive regulator of bone growth. In the present study, we infused CNP into uremic rats and observed whether CNP could attenuate ROD through the inhibition of FGF-23 cascades. In uremic rats, CNP administration significantly alleviated renal dysfunction, calcium phosphate metabolic disorders, hypovitaminosis D, secondary hyperparathyroidism, the decrease in bone turnover markers and retarded bone pathological progression. More importantly, within FGF-23/mitogen-activated protein kinase (MAPK) signaling, the fibroblast growth factor receptor-1, Klotho and alternative (STAT-1/phospho-STAT-1) elements were upregulated by CNP, whereas FGF-23, RAF-1/phospho-RAF-1, and downstream (ERK/phospho-ERK and P38/phospho-P38) elements were paradoxically underexpressed in bone tissue. Therefore, CNP exerts a therapeutic effect on ROD through inhibition of FGF-23/MAPK signaling at the RAF-1 level.

## Bone disease: Treating a complication of chronic kidney disease

A naturally occurring peptide (a small protein) might be useful for treating renal osteodystrophy (ROD), a bone disease common in people with chronic kidney disease. ROD develops when kidney disease disrupts the normal metabolism of calcium, phosphate and vitamin D essential to healthy bones. Peng Hu and colleagues at the First Affiliated Hospital of Anhui Medical University, Hefei, China, found that administering ‘C-type natriuretic peptide’ intravenously to rats which had been surgically and chemically treated to induce a condition modelling ROD in humans resulted in improvement. The therapeutic effect of the peptide was mediated by inhibiting a molecular signaling pathway which leads to the over-production of a specific fibroblast growth factor protein in bone cells in ROD. The significance of these results for humans should be investigated further.

## Introduction

Renal osteodystrophy (ROD) is a common skeletal complication of chronic kidney disease (CKD) due to abnormal metabolisms of calcium (Ca), phosphate (P), and vitamin D, and their complex interactions with parathormone (PTH)^1^. The gold standard for ROD diagnosis is a bone biopsy, which is used to acquire morphologic information on bone remodeling, osteoid accumulation, and fibrosis degree^2^. In a previous study, Wesseling-Perry et al. enrolled 52 CKD patients between the ages of 2 and 21 years, and found that excess osteoid accumulation was identified in 43% of patients with CKD stage 2 and in 86% of patients with CKD stage 4/5; consistently, prolongation in osteoid maturation time was observed in 43% of patients with stage 2 CKD, 79% with CKD stage 3, and 79% with CKD stage 4/5. In general, ROD occurs as early as CKD stage 2 and seems ubiquitous in almost all pediatric patients with CKD stage 5^3^. Furthermore, recent evidence indicates that mineral and bone disorders are associated with an increased risk of fracture. A prospective study based on the Taiwan Health Insurance Dataset showed that the cumulative incidence of orthopedic fracture was 59.6‰ from 2004 to 2008, and the annual incidence of orthopedic fracture in CKD patients was at least 1.72-fold higher than that in the general population^4^. However, bone biopsy is an invasive tool for ROD diagnosis, and moreover, few clinical centers are familiar with this technique^5^. Despite many advances in the exploration of noninvasive biomarkers and the stimulation of novel therapeutic regimens, ROD continues to be a major diagnostic and therapeutic challenge for the practicing nephrologist.

Fibroblast growth factor (FGF)-23, primarily isolated from human osteoblasts, exerts an important hormonal regulatory effect on circulating phosphate and calcitriol through suppressing the expression of the Na/Pi IIa and IIc cotransporters in the brush border of the proximal tubule^6,7^. In the concurrent presence of FGF receptor (FGFR)-1 and the transmembrane protein Klotho, FGF-23 also antagonizes 1α-hydroxylase, subsequently resulting in 1, 25-dihydroxyvitamin D deficiency and calcium malabsorption, which may initiate the development of secondary hyperparathyroidism^8^. FGF-23 is not only derived from osteoblasts but also can regulate their proliferation and maturation. In 2004, Sitara et al. generated a FGF-23 null mouse model and observed marked skeletal abnormalities and decreased bone mineral density^9^. Several years later, Wang et al. transfected human FGF-23 into osteoblasts in vitro, and found that overexpressed FGF-23 could significantly disturb osteoblast differentiation and matrix mineralization^10^. Similarly, Murali and colleagues established a murine model of X-linked hypophosphatemia and showed that increased serum FGF-23 was associated with widened osteoid seams and enlarged osteocyte lacunae in histological bone^11^. In addition, a growing body of literature has documented that elevated FGF-23 levels are a common feature of CKD. In uremic mice, the mean serum FGF-23 concentration was 900.9 ± 87.4 pg/ml, a value 6.7-fold higher than baseline values of 134.5 ± 15.8 pg/ml^12^. In agreement with the above animal study, Gutierrez et al. revealed that circulating FGF-23 increased as early as CKD stage 2 with a value of 86.2 ± 61.4 RU/ml. Since then, FGF-23 has been shown to be inversely related to estimated glomerular filtration rate (GFR); by the time patients reach end stage, the level of FGF-23 is almost five-fold above stage 2 levels^13^. However, there is scant information on the involvement of FGF-23 in ROD pathogenesis.

C-type natriuretic peptide (CNP) was first purified from central nervous tissues in 1990^14^. During the last two decades, this peptide has been identified in vascular endothelial, cardiac, renal, skeletal, and reproductive tissues^15,16^. CNP specifically binds to natriuretic peptide receptor (NPR)-B and subsequently causes a soaring increase in the synthesis of intracellular cyclic guanosine monophosphate (cGMP). On the other hand, it is degraded by three main mechanisms: neutral endopeptidase (NEP), NPR-C, and urinary excretion^17^. Distinct from FGF-23, CNP stimulates the proliferation, differentiation, and mineralization of osteoblast-like cells^18,19^. In addition, CNP acts as a potent positive regulator of endochondral bone growth. To date, at least 29 unique inactivating missense mutations in NPR-B have been identified in human dwarfism^20^. In contrast, overexpression of CNP can significantly improve the impairment in longitudinal growth of CNP knockout mice^21^. Encouragingly, there has been a substantial breakthrough in the knowledge of CNP metabolism in nephropathies. Our recent study published in *Peptides* demonstrated that both urinary excretion and renal expression of CNP are enhanced in the early stage of CKD, whereas they gradually decline with CKD progression^22^. On the basis of the evidence presented above, CNP decline may contribute, in part, to the onset of ROD. Moreover, whether CNP supplementation could be a promising strategy for the treatment of ROD is not well elucidated.

Because the effects of FGF-23 and CNP on bone formation appear to oppose each other, it is reasonable to propose a direct interaction of their signaling pathways. In the present study, we continuously infused exogenous CNP into uremic rats and observed whether CNP could attenuate ROD through the inhibition of FGF-23 cascades.

## Materials and methods

### Animals and treatment

Male Sprague-Dawley rats weighting 120–150 g were housed at an ambient temperature of 23 ± 1 °C and exposed to a daily 12 h light/dark cycle (lights on 07:00 to 19:00 h) with free access to tap water and a pellet diet. Animals were treated humanely using approved procedures in accordance with the guidelines of the Institutional Animal Use and Care Committee of Anhui Medical University. The fasted animals were operated under intraperitoneal pentobarbital anesthesia (60 mg/kg body weight) and sterile conditions. The operation was performed in accordance with the permission of our institution’s Local Medical Ethics Committee (No. LLSC20150009). The uremic rat model was induced by nephrectomy of the left kidney plus intravenous injection of adriamycin (5 mg/kg body weight, Wanle Pharma Ltd., Shenzhen, China) dissolved in 0.9% saline. Rats were divided randomly into the uremic group and CNP-treated group. Forty-eight rats were used in each group, and we added additional rats immediately if any rats were excluded from our study because of death. The total mortality rate was 18.2% (32/176); for each group, the mortality was 14.3% (8/56) in the sham-operated group, 21.3% (13/61) in the uremic group, and 18.6% (11/59) in the CNP-treated group. The CNP-treated group was given a continuous infusion of CNP (0.05 μg/kg/min × 1 h, 1 μg/kg/min = 0.5 nM/kg/min, Sangon Biotech, Shanghai, China) through the caudal vein from the day of model induction until the day of sacrifice^23^. The continuous infusion was performed by a Microinfusion Pump (single track Wz-50C6, Zhejiang University Medical Instrument Co., Ltd., Zhejiang, China). The other 48 animals in the sham-operated group underwent a sham laparotomy with ureteric manipulation through a midline incision and given a continuous infusion of 0.9% saline through the caudal vein from the day of operation until the day of sacrifice. Subsequently, animals in each group were separated into eight experimental subgroups (*n* = 6), anesthetized by intraperitoneal pentobarbital injection, and sacrificed by heart puncture at 24, 72 h, 1, 2, 3 weeks, 1, 2, and 3 months after operation. On the basis of the disease duration, the eight subgroups were divided into acute stage, progressive stage, and chronic stage (acute stage: disease duration≤1 week; progressive stage: 1 week < disease duration ≤ 1 month; chronic stage: 1 month < disease duration ≤ 3 months).

### Laboratory analysis

All rats were individually placed in metabolic cages, and urine was collected for 24 h. The urine creatinine concentration (UCr) and urinary protein concentration (UP) from 24 h urine samples were determined by a biuret colorimetric method (Boehringer Mannheim, Italy). Blood samples were taken from the abdominal aorta and centrifuged at 1600 × *g* for 15 min at 4 °C. Serum albumin (Alb), blood urea nitrogen (BUN), serum creatinine (SCr), uric acid (UA), calcium, and phosphate were measured by standard enzymatic method (Randox, UK). The protein levels of serum 25-hydroxyvitamin D [25-(OH) D], parathyroid hormone (PTH), osteocalcin (OC), bone-specific alkaline phosphatase (bAP), total alkaline phosphatase (tAP), procollagen type I carboxy-terminal propeptide (PICP), cross-linked carboxyterminal telopeptide of type I collagen (ICTP), tartrate-resistant acid phosphatase (TRAP), urinary deoxypyridinoline (DPD), and pyridinoline (PYD) were determined using commercially available enzyme-linked immunosorbent assay (ELISA) kits (Invitrogen, Carlsbad, USA) according to the manufacturer’s protocols.

### Renal morphology

At harvest, each kidney was washed with saline and blotted dry on gauze. Midcoronal kidney sections were fixed in 4% paraformaldehyde and embedded in paraffin. Paraffin sections (4 μm thick) were stained with hematoxylin and eosin. Renal morphologic lesions were evaluated in 10 randomly selected, nonoverlapping specimens from each rat at ×400 magnification.

### Bone morphology

At harvest, the distal femurs were excised, placed in 70% ethanol, and dehydrated in increasing concentrations of ethanol. Specimens were embedded in methyl methacrylate and not decalcified. Bones were sectioned longitudinally through the frontal plane in 4 μm sections using a microtome (Leica, Wetzlar, Germany) and stained with Goldner’s trichrome for trabecular and cellular analysis. Changes in osteoblast, osteoclast, osteoid volume, and trabecular thickness were observed at ×400 magnification.

### Real-time (RT)-PCR

Total RNA was extracted from bone tissues using TRIzol reagent (Invitrogen, USA). Ultraviolet spectrophotometry to measure absorbance and agarose gel electrophoresis confirmed that there was no degradation of RNA. One microgram of total RNA was reverse transcribed into cDNA using an ExScript RT reagent kit (Takara Biotechnology, Dalian, China). The primers for FGF-23, Klotho, FGFR-1, signal transducers and activators of transcription (STAT-1), RAF-1, extracellular signal-regulated kinases (ERK), P38, collagen (Col) X, and glyceraldehyde-3-phosphate dehydrogenase (GAPDH) are shown in Table 1. Real-time PCR was undertaken with an ABI 7900 sequence detection system (Applied Biosystems, Foster City, USA) using SYBR Green PCR Master Mix (Applied Biosystems, Foster City, USA) in accordance with the manufacturer’s instructions. Amplification conditions included predenaturation at 95 °C for 2 min; then 40 cycles of denaturation at 94 °C for 20 s, annealing at 60 °C for 20 s, extension at 72 °C for 30 s; and finally one cycle of 5 min at 72 °C. Relative expression was calculated using the 2^−ΔΔCt^ method by normalization to GAPDH housekeeping gene expression and presented as fold increase relative to control^24^.

**Table 1:** List and sequence of primers

Variable	Sequence	Product length (bp)
*FGF-23*		
Sense	5′-GATGCTGGCTCCGTAGTGATAAT-3′	164
Antisense	5′-TGATGCTTCGGTGACAGGTAGA-3′	
*Klotho*		
Sense	5′-GCCGAGCAAGACTCACTG-3′	101
Antisense	5′-GCAAAGTAGCCACAAAGGT-3′	
*FGFR-1*		
Sense	5′-GGCACCTGAGGCATTGTTT-3′	170
Antisense	5′-TACTGGGCTTGTCCATTCG-3′	
*STAT-1*		
Sense	5′-GGACGTTCCTGCTTAGGT-3′	121
Antisense	5′-TTCTTCGTGTAGGGCTCA-3′	
*RAF-1*		
Sense	5′-TGTTTGATGGCTCCAGTT-3′	193
Antisense	5′-GCTTTCATAAGGCAGTCG-3′	
*ERK*		
Sense	5′-CGGATTGCTGACCCTGAG-3′	111
Antisense	5′-GGATTTGGTGTAGCCCTTG-3′	
*P38*		
Sense	5′-GGACCTAAAGCCCAGCAA-3′	186
Antisense	5′-CAGCCCACGGACCAAATA-3′	
*Col-X*		
Sense	5′-TCTGGGATGCCTCTTGTC-3′	155
Antisense	5′-GATCTTGGGTCATAGTGCTG-3′	
*GAPDH*		
Sense	5′-GTTACCAGGGCTGCCTTCTC-3′	168
Antisense	5′-GGGTTTCCCGTTGATGACC-3′	

### Immunofluorescence staining

Immunofluorescence was performed on 4 μm paraffin-embedded sections of distal femoral epiphyses. All sections were blocked with 500 μl of blocking solution [3% (w/v) bovine serum albumin with 0.2% (v/v) Triton X-100 in phosphate-buffered saline (PBS)] at room temperature for 1 h followed by incubation with anti-FGF-23, Klotho, FGFR-1, STAT-1, RAF-1, ERK, P38, and Col X antibodies (1:50, MDL, Beijing, China) at 4 °C overnight. Sections were washed with PBS and incubated with Alexa Fluor 488-labeled goat anti-rabbit immunoglobulin G (1:200; Jackson ImmunoResearch, USA) at room temperature for 1 h in a dark chamber followed by counterstaining of nuclei with 4′,6-diamidino-2-phenylindole (DAPI) (0.1 μg/ml) for 10 min. Sections were washed with PBS, and immunofluorescence images were captured with a Leica DM3000 confocal microscope (Leica, Wetzlar, Germany).

### Western blotting analysis

Bone tissues were homogenized in ice-cold buffer (40 mM KCl, 10 mM Hepes, pH 7.9, 3 mM MgCl_2_, 5% glycerol, 0.5 μg/ml leupeptin, 0.1 μg/ml aprotinin, 1.5 μg/ml pepstatin, and 100 mg/ml phenylmethylsulphonyl fluoride) with a Polytron homogenizer for 15–20 s. The homogenates were centrifuged at 500 × *g* for 10 min at 41 °C, and the supernatants were recentrifuged at 12,000 × *g* for 15 min at 41 °C. The pellets were resuspended in 0.5 ml homogenizing buffer containing 0.5% Nonidet P-40 (Boster, Wuhan, China), and the total protein concentration of the supernatant was determined by the dyebinding method using bovine serum albumin as the standard. Samples (50 μg) were subjected to 10% sodium dodecyl sulfate–polyacrylamide gel electrophoresis and transferred onto a nitrocellulose membrane (Bio-Rad, Beijing, China). The membranes were blocked with Tris-buffered saline and 0.1% (w/v) Tween 20 (TBST) containing 5% defatted dry milk (Bio-Rad, Beijing, China) overnight. Immunoblots were incubated with anti-FGF-23, Klotho, FGFR-1, STAT-1, phospho-STAT-1, RAF-1, phospho-RAF-1, ERK, phospho-ERK, P38, phospho-P38, Col X, and GAPDH primary antibodies (Medical Discovery Leader, Beijing, PR China) at 4 °C overnight. The membranes were washed three times (10 min each) with TBST, followed by incubation with horseradish peroxidase-conjugated anti-rabbit immunoglobulin G antibodies (1:3000 dilution; Medical Discovery Leader, Beijing, PR China) at room temperature for 60 min. The reaction products were detected using the enhanced chemiluminescence detection system and exposed on radio-graphic films for variable periods.

### Statistical analyses

All values are expressed as the mean ± SEM. The interaction between treatment and time on renal function, calcium phosphate metabolism, bone formation, FGF-23/MAPK signaling, and Col X expression were determined using two-way ANOVA and post hoc Fisher’s least significant difference (LSD) test; Bonferroni correction was applied to the results of post hoc independent *t*-tests. All values were two sided, and *P* < 0.05 was considered to indicate significance. Statistical analyses were performed using the Statistical Package for the Social Sciences (SPSS) version 17.0.

## Results

### The establishment of the ROD model in rats

**Fig. 1:**
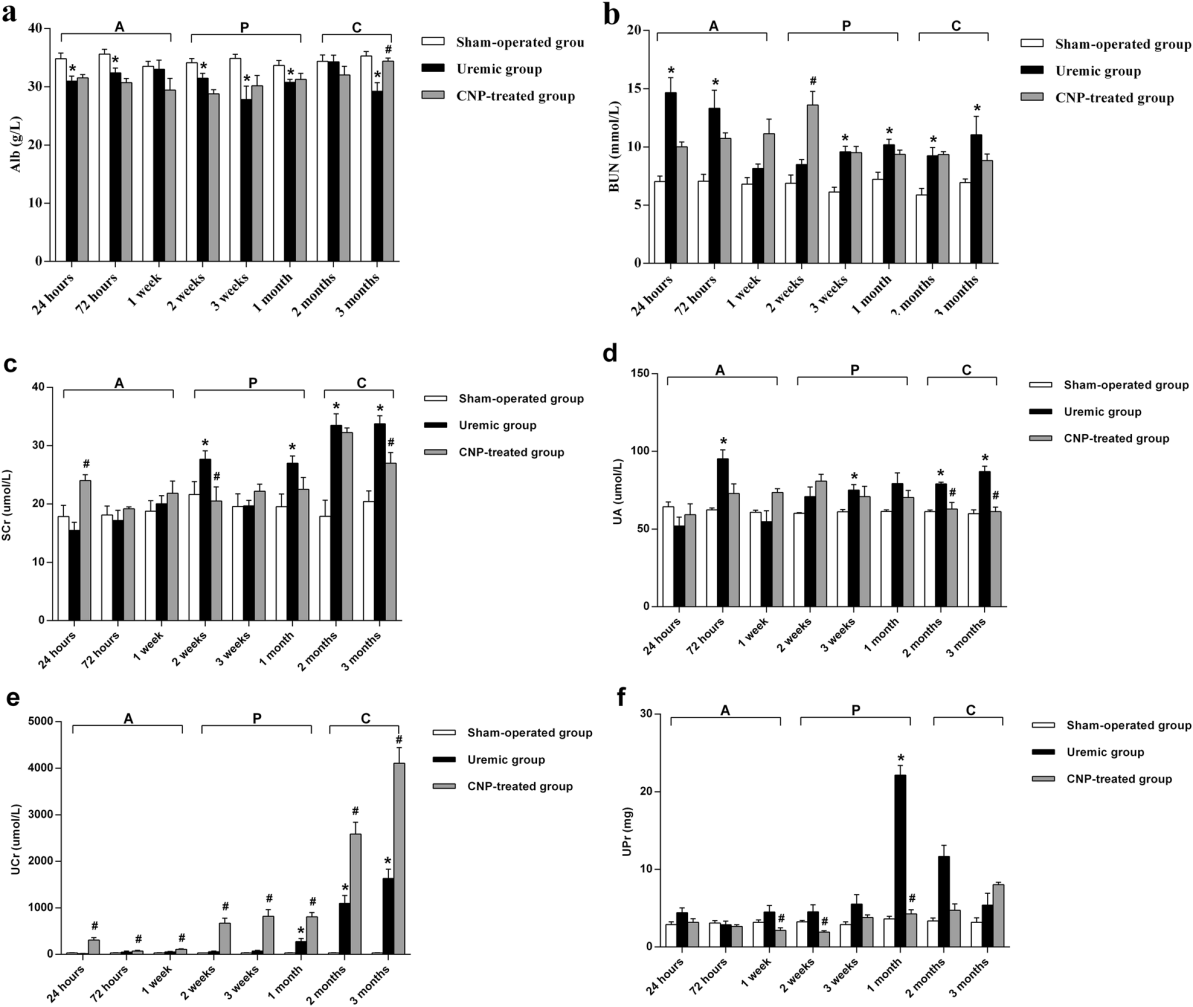
Renal function indices in sham-operated, uremic and CNP-treated rats. Uremic group vs. sham-operated group**:** Serum Alb in the uremic group was significantly decreased throughout the observational period (**a**); serum BUN and UA in the uremic group were significantly higher in every stage (**b**, **d**); SCr, UCr, and UPr were elevated in the progressive stage and chronic stage (**c**, **e**, and **f**). CNP-treated group vs. uremic group**:** hypoalbuminemia in the uremic group could be dramatically reversed by CNP until 3 months (**a**); SCr, UA, and BUN in the CNP-treated group were markedly higher at 24 h, 1 and 2 weeks posttreatment, respectively, whereas SCr was significantly decreased in both the progressive stage and chronic stage, and UA was significantly decreased only in the chronic stage (**b**–**d**); The CNP-treated group showed a dramatic elevation in UCr at each time point (**e**); CNP administration resulted in a prominent reduction in UPr from the acute stage to the progressive stage (**f**). CNP-treated group vs. sham-operated group**:** the levels of Alb, SCr, UA, and UPr in the CNP-treated group gradually became similar to those in the sham-operated group at 3 months postoperatively (**a**, **b**, **d**, and **f**). *P < 0.05, significantly different from the corresponding sham-operated group; ^#^*P* < 0.05, significantly different from the corresponding uremic group. A acute stage, P progressive stage, and C chronic stage

**Fig. 2:**
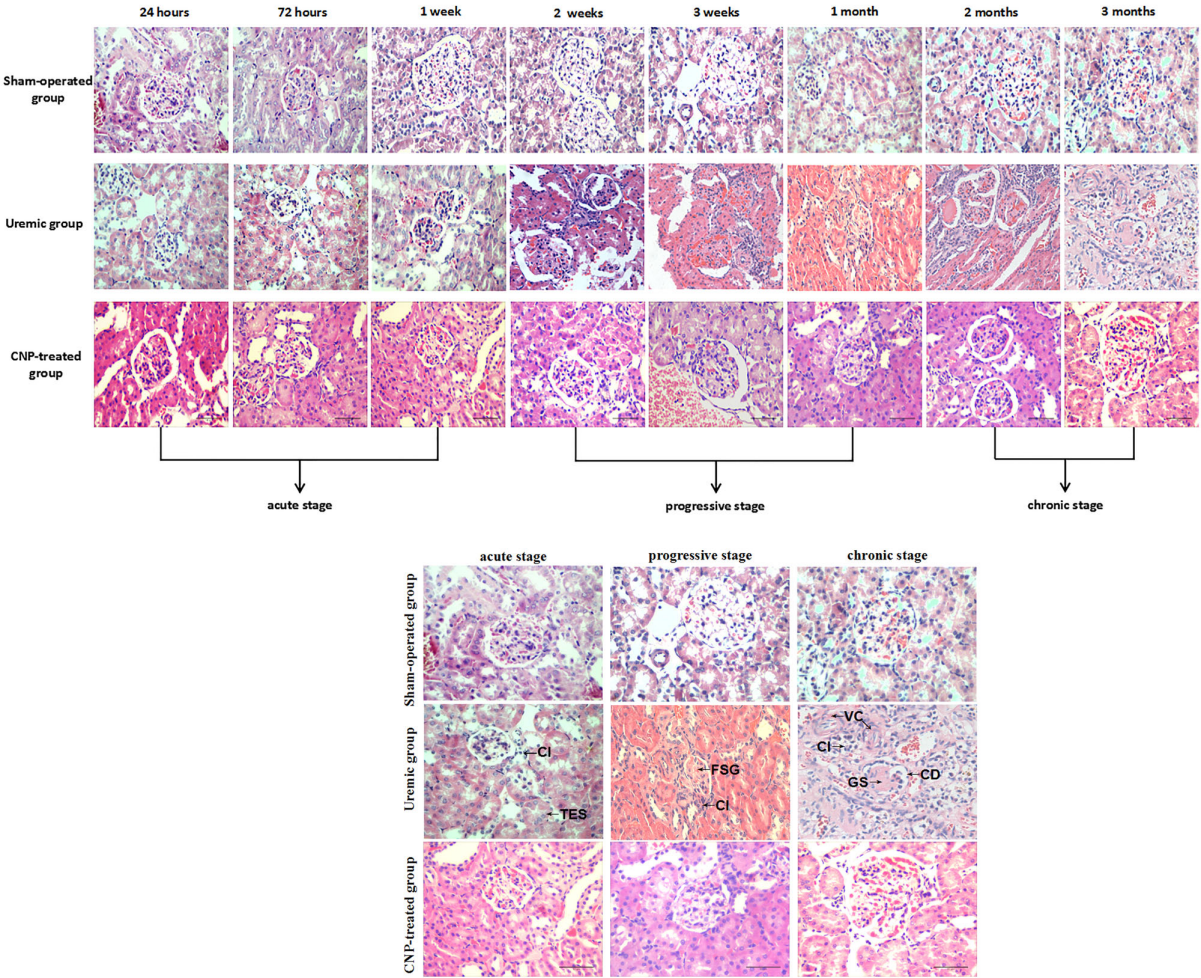
Representative histological images of kidneys in sham-operated, uremic and CNP-treated rats. Sham-operated group**:** Sections derived from sham-operated kidneys had a normal appearance. Uremic group**:** In the acute stage, renal damage was limited to renal tubular epithelial swelling (TES), and interstitial expansion, accompanied by inflammatory cell infiltration (CI) in both the glomeruli and interstitium; subsequently, the uremic group displayed focal segmental glomerulosclerosis (FSG), inflammatory cell infiltration (CI), and marked interstitial expansion during the progressive stage; when entering into the chronic stage, renal pathology was characterized by glomerulosclerosis (GS), corpuscle distortion (CD), inflammatory cell infiltration (CI), and vascular congestion (VC). In contrast, sections derived from sham-operated kidneys had a normal appearance. Therefore, adriamycin injection could induce persistent renal dysfunction and pathological lesions in rats. CNP-treated group: There were no apparent differences in any aspects of renal damage between the CNP-treated group and uremic group in the acute stage. In the progressive stage and chronic stage, the degree of focal segmental glomerulosclerosis (FSG), corpuscle distortion (CD), and interstitial expansion in the uremic group was prominently ameliorated by CNP

Two-way ANOVA with a post hoc Fisher’s LSD test revealed that adriamycin had a significant effect on the bone length of femurs (*t* = 2.6, *P* = 0.017). In the uremic group, the bone length of femurs was significantly shorter than that in the sham-operated group at 3 months postoperation (uremic group: 3.45 ± 0.05 cm vs. sham-operated group: 3.59 ± 0.01 cm) (*P* < 0.05). Renal function indices of the uremic group and sham-operated group are shown in Fig. 1. The results from the LSD test showed that adriamycin significantly affected renal function (*t* ≥ 4.00, *P* < 0.001). Renal function indices are showed in the Supplemental Table in detail. In comparison with the corresponding parameters in the sham-operated group, BUN and UA in the uremic group were significantly higher in every stage (both *P* < 0.05) (Fig. 1b, d), and SCr, UCr, and UPr were elevated in the progressive stage and chronic stage (all *P* < 0.05) (Fig. 1c, e, f). Nevertheless, Alb in the uremic group was significantly lower than that in the sham-operated group throughout the observational period (*P* < 0.05) (Fig. 1a). Figure 2 shows representative light microscopy images of the renal cortex in each group. Renal pathology in the uremic group gradually became aggravated. In the acute stage, renal damage was limited to renal tubular epithelial swelling and interstitial expansion, accompanied by an inflammatory cell infiltration in both the glomeruli and interstitium; subsequently, the uremic group displayed focal segmental glomerulosclerosis, inflammatory cell infiltration, and marked interstitial expansion during the progressive stage; when entering into the chronic stage, renal pathology was characterized by glomerulosclerosis, corpuscle distortion, inflammatory cell infiltration, and vascular congestion. In contrast, sections derived from sham-operated kidneys had a normal appearance. Therefore, adriamycin injection could induce persistent renal dysfunction and pathological lesions in rats.

**Fig. 3:**
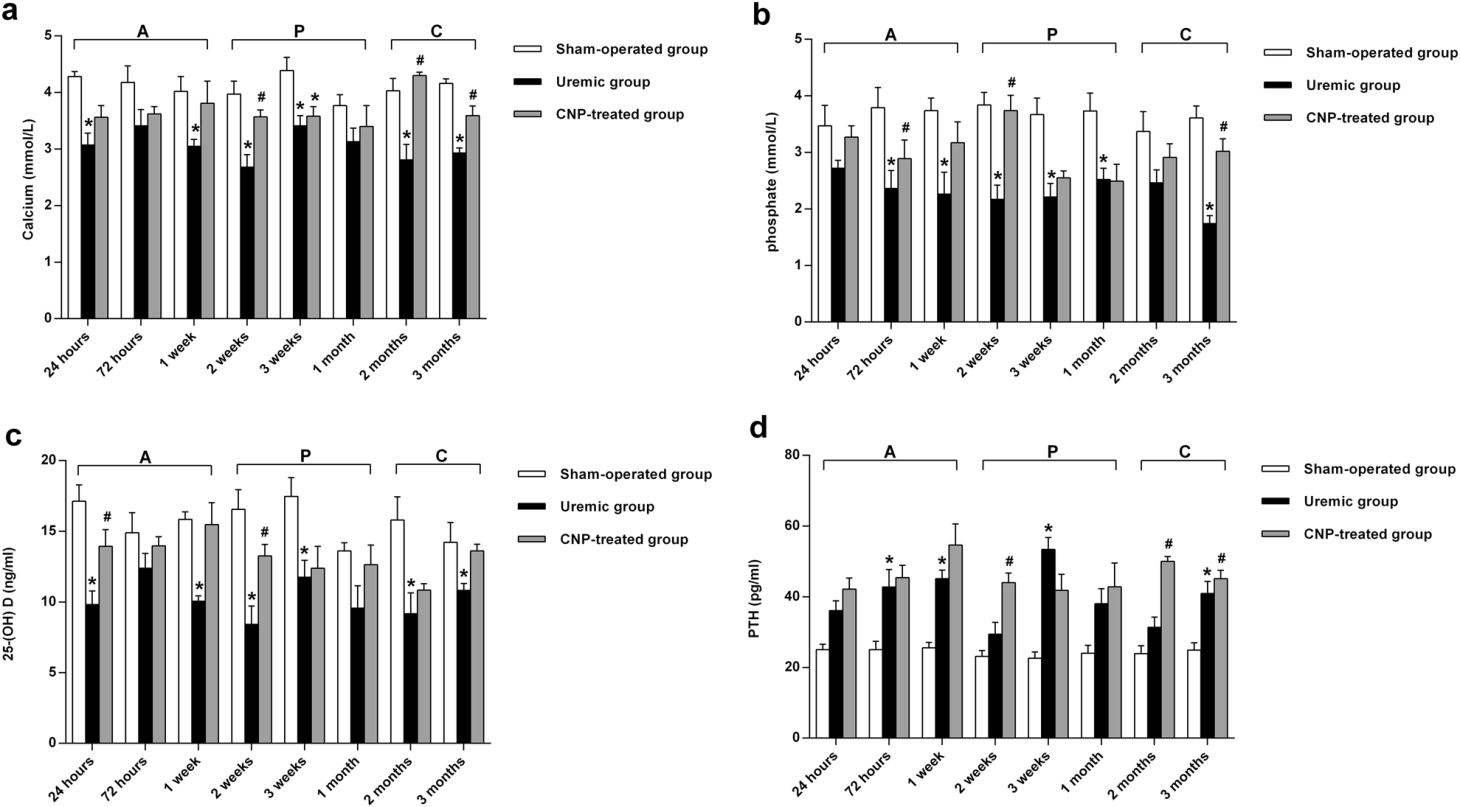
Serum calcium, phosphate, 25-(OH) D, and PTH in sham-operated, uremic, and CNP-treated rats. Uremic group vs. sham-operated group**:** The levels of calcium, phosphate, and 25-(OH) D gradually decreased during our observational period and moreover, were significantly lower than those in the sham-operated group in every stage (**a**–**c**); serum PTH was significantly higher in the uremic group than in the sham-operated group from the acute stage to the chronic stage (**d**). CNP-treated group vs. uremic group: CNP administration could significantly enhance calcium and PTH levels in the progressive stage and chronic stage (**a**, **d**); 25-(OH) D in the acute stage and progressive stage (**c**); and phosphate throughout our observational period (**b**). CNP-treated group vs. sham-operated group: CNP recovered the levels of calcium, phosphate, and 25-(OH) D to those in the corresponding sham-operated group in the chronic stage (**a**–**c**); PTH significantly increased in the CNP-treated group throughout the whole observational period (**d**). ^*^P < 0.05, significantly different from the corresponding sham-operated group; ^#^*P* < 0.05, significantly different from the corresponding uremic group. A acute stage, P progressive stage, and C chronic stage

**Fig. 4:**
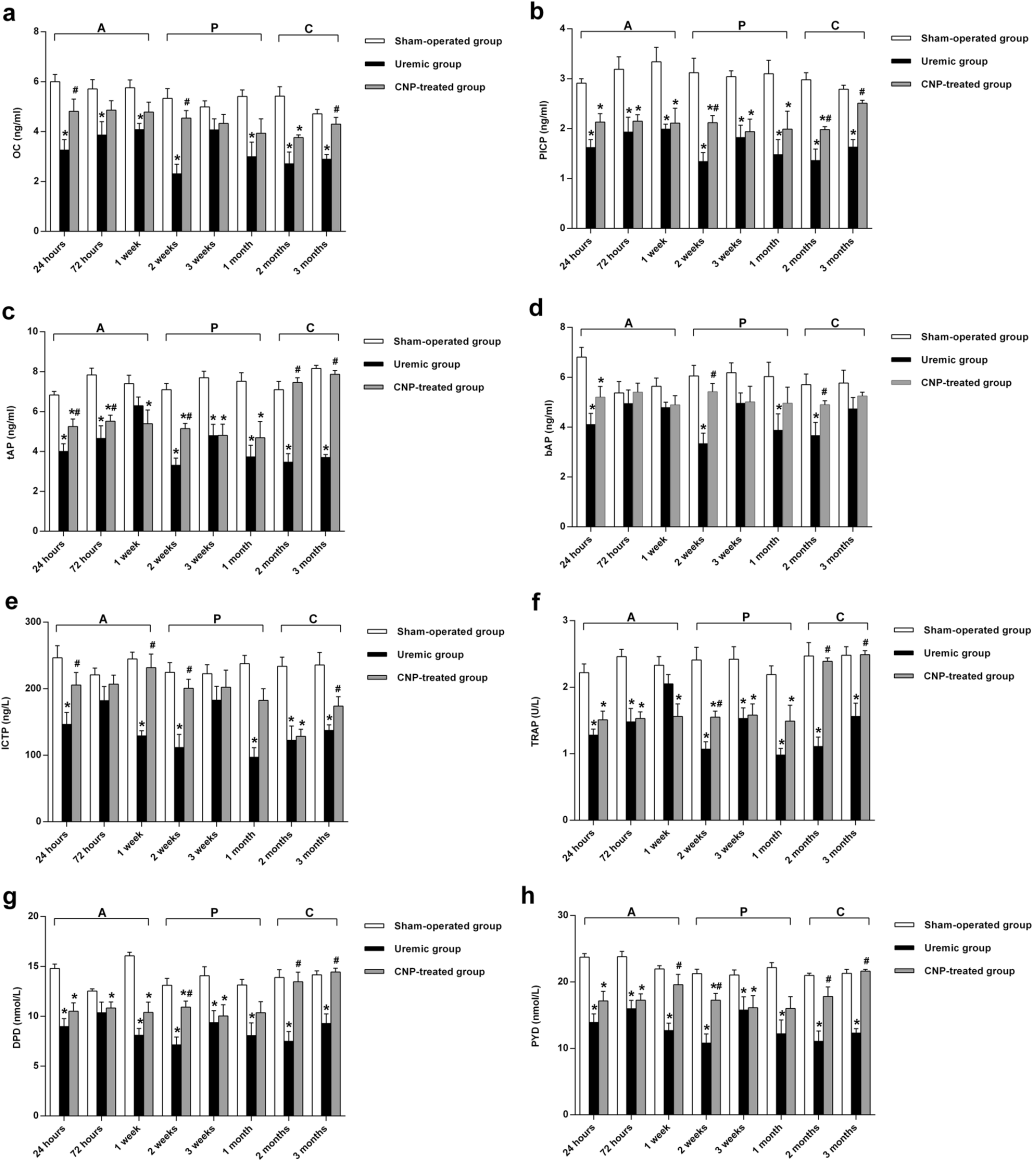
Biochemical markers of bone turnover in sham-operated, uremic, and CNP-treated rats. Uremic group *vs* sham-operated group: Osteoblastic (OC, PICP, tAP, and bAP) and osteolytic (ICTP, TRAP, PYD, and DPD) proteins were remarkably different between the two groups, and the uremic group developed significantly lower levels of bone turnover markers during this study (**a**–**h**); CNP-treated group vs. uremic group: Significant higher levels of OC, tAP, ICTP, and PYD were noted in the CNP-treated group in each stage (**a**, **c**, **e**, **h**); PICP, bAP, TRAP, and DPD increased significantly in the CNP-treated group from the progressive stage to the chronic stage (**b**, **d**, **f**, **g**). CNP-treated group vs. sham-operated group**:** in the CNP-treated group, the levels of osteoblastic (OC, PICP, tAP, and bAP) and osteolytic (TRAP, PYD, and DPD) biomarkers eventually increased to levels comparable to those in the sham-operated group at 3 months postoperatively (**a**–**d**, **f**–**h**); the ICTP level was significantly lower than that in the sham-operated group in the chronic stage (**e**).^*^P < 0.05, significantly different from the corresponding sham-operated group; ^#^*P* < 0.05, significantly different from the corresponding uremic group. A acute stage, P progressive stage, and C chronic stage

**Fig. 5:**
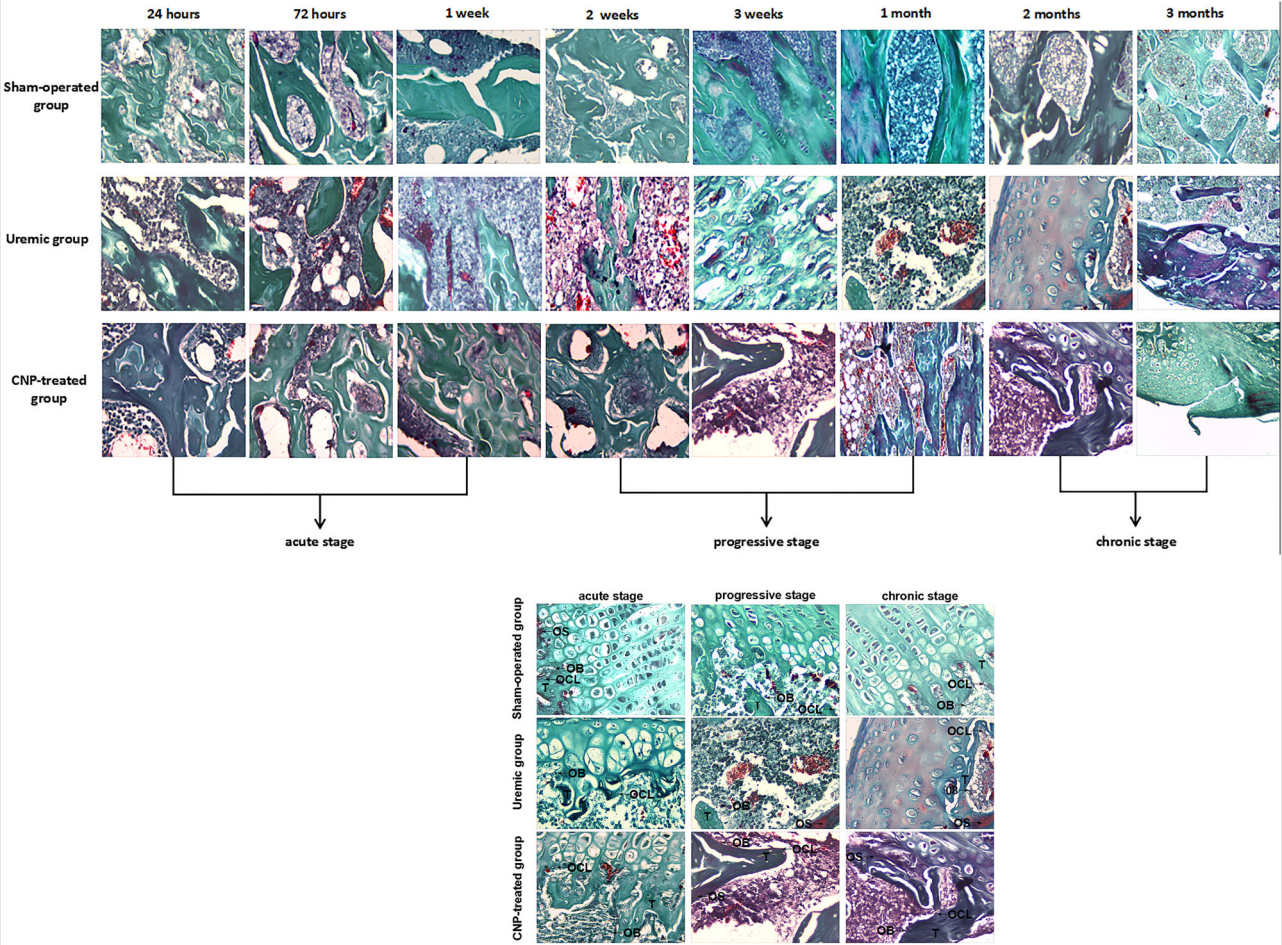
Goldner’s trichrome staining of distal femurs in sham-operated, uremic, and CNP-treated rats. Uremic group vs. sham-operated group: In the acute stage, there was no dramatic difference in bone histology between the uremic group and sham-operated group. Pathological lesions in the uremic group formed in the progressive stage and gradually deteriorated thereafter. Adriamycin injection triggered a low-turnover bone lesion characterized by an evident decrease in osteoblasts (OB), osteoclasts (OCL), and trabecular (T) volume thickness, and a clear elevation in osteoid (OS) volume, compared with the sham-operated group. CNP-treated group vs. uremic group: In the acute stage, bone histology of the CNP-treated group was identical to that of the uremic group. Subsequently, CNP injection contributed to prominent elevations in osteoblasts (OB), osteoclasts (OCL), osteoid (OS) volume, and trabecular (T) thickness in the uremic group

The changes in serum calcium, phosphate, 25-(OH) D, and PTH are described in Fig. 3. Two-way ANOVA with a post hoc Fisher’s LSD test revealed that adriamycin had a significant effect on serum calcium, phosphate, 25-(OH) D, and PTH (*t* ≥ 8.12, *P* < 0.001). In the uremic group, the levels of calcium, phosphate, and 25-(OH) D gradually declined during our observational period, and moreover, were significantly lower than those in their sham-operated counterparts in every stage (all *P* < 0.05) (Fig. 3a–c); in contrast, serum PTH was significantly higher in the uremic group from the acute stage to the chronic stage than in the sham-operated group (*P* < 0.01) (Fig. 3d). Biochemical markers of bone turnover in the uremic group and sham-operated group are presented in Fig. 4. The results from the LSD test showed that adriamycin significantly affected osteoblastic and osteolytic proteins (*t* ≥ 6.6, *P* < 0.001). Osteoblastic (OC, PICP, tAP, and bAP) (Fig. 4a–d) and osteolytic (ICTP, TRAP, PYD, and DPD) (Fig. 4e–h) protein levels were remarkably different between the two groups, and the uremic group developed significantly lower levels of bone turnover markers relative to the sham-operated group during this study (all *P* < 0.05). Representative histological images of distal femurs are shown in Fig. 5. In the acute stage, there was no dramatic difference in bone histology between the uremic group and sham-operated group. Pathological lesions in the uremic group initiated in the progressive stage and gradually deteriorated thereafter. Adriamycin injection triggered a low-turnover bone lesion characterized by an evident decrease in osteoblasts, osteoclasts, and trabecular volume thickness and a noticeable elevation in osteoid volume in the uremic group compared with the sham-operated group.

### Therapeutic effect of CNP on ROD

Two-way ANOVA indicated that the bone length of femurs was significantly affected by the main effects of treatment (CNP) (*F*_treatment_ = 3.98, *P* = 0.021) and time (*F*_time_ = 172.58, *P* < 0.001); however, the interaction between treatment and time was not significant (*F*_interaction_ = 0.80, *P* = 0.673). Post hoc analyses demonstrated that there was no significant difference in bone length of femurs between the CNP-treated group and uremic group (*P* > 0.05), whereas the bone length of femurs in the CNP-treated group was significantly shorter than that in the sham-operated group at 3 months postoperatively (*P* < 0.01). Renal function indices of the CNP-treated group and uremic group are presented graphically in Fig. 1. There were significant main effects of treatment (*F*_treatment_ ≥ 11.80, *P* < 0.001) and time (*F*_time_ ≥ 2.68, *P* < 0.05) and a significant interaction effect of treatment and time (*F*_interaction_ ≥ 2.12, *P* < 0.05) on renal function. The CNP-treated group showed a dramatic elevation in UCr levels relative to the corresponding uremic group at each time point (*P* < 0.05) (Fig. 1e). Compared with the same parameters in the uremic group, SCr, UA, and BUN in the CNP-treated group were markedly higher at 24 h, 1 and 2 weeks posttreatment, respectively (all *P* < 0.05), whereas SCr was significantly decreased in both the progressive stage and chronic stage (*P* < 0.01). UA was significantly decreased only in the chronic stage (*P* < 0.001) (Fig. 1b–d). CNP administration resulted in a prominent reduction in UPr from the acute stage to the progressive stage (*P* < 0.05) (Fig. 1f). Moreover, hypoalbuminemia in the uremic group could be dramatically reversed by CNP until 3 months (*P* < 0.05) (Fig. 1a). The levels of Alb, SCr, UA, and UPr in the CNP-treated group eventually became similar to those in the sham-operated group at 3 months post operation (all *P* > 0.05) (Fig. 1). Representative histological images of kidneys are shown in Fig. 2. There were no apparent differences in any aspects of renal damage between the CNP-treated group and uremic group in the acute stage. In the progressive stage and chronic stage, the degree of focal segmental glomerulosclerosis, corpuscle distortion, and interstitial expansion of the uremic group were prominently ameliorated by CNP.

The changes in serum calcium, phosphate, 25-(OH) D, and PTH in the CNP-treated group and uremic group are presented in Fig. 3. A significant main effect of treatment (*F*_treatment_ ≥ 32.94, *P* < 0.001) was found on serum calcium, phosphate, and 25-(OH) D, and significant main effects of treatment (*F*_treatment_ = 74.71, *P* < 0.001) and time (*F*_time_ = 2.17, *P* < 0.05) and a significant interaction between treatment and time (*F*_interaction_ = 1.95, *P* < 0.05) were observed for PTH. CNP administration could significantly enhance calcium and PTH levels in the progressive stage and chronic stage (Fig. 3a, d), 25-(OH) D in the acute stage and progressive stage (Fig. 3c), and phosphate throughout our observational period (all *P* < 0.05) (Fig. 3b). In the chronic stage, CNP corrected the levels of calcium, phosphate, and 25-(OH)D to the levels observed in the corresponding sham-operated group, whereas PTH was significantly increased in the CNP-treated group throughout the whole observational period (*P* < 0.05) (Fig. 3). Biochemical markers of bone turnover in the CNP-treated group and uremic group are presented in Fig. 4. It is evident that there was a significant main effect of time on OC, tAP, ICTP, TRAP, and PYD (*F*_time_ ≥ 2.60, *P* < 0.05); a significant main effect of treatment on osteoblastic and osteolytic proteins (*F*_treatment_ ≥ 22.12, *P* < 0.001); and a significant interaction effect between treatment and time on tAP, ICTP, TRAP, PYD, and DPD (*F*_interaction_ ≥ 2.09, *P* < 0.05). Significantly higher levels of OC, tAP, ICTP, and PYD were noted in the CNP-treated group in each stage (all *P* < 0.05) (Fig. 4a, c, e, h); however, PICP, bAP, TRAP, and DPD increased significantly in the CNP-treated group from the progressive stage to the chronic stage (all *P* < 0.05) (Fig. 4b, d, f, g). In the CNP-treated group, the levels of osteoblastic (OC, PICP, tAP, and bAP) and osteolytic (TRAP, PYD, and DPD) biomarkers eventually increased to the levels of the sham-operated group at 3 months postoperatively (all *P* > 0.05), whereas ICTP was significantly lower in the chronic stage than in the sham-operated group (*P* < 0.05) (Fig. 4). Representative histological images of distal femurs of the CNP-treated group and uremic group are displayed in Fig. 5. In the acute stage, bone histology of the CNP-treated group was identical to that of the uremic group. Subsequently, CNP injection contributed to prominent elevations in osteoblasts, osteoclasts, and trabecular thickness in the uremic group.

### FGF-23/mitogen-activated protein kinase (MAPK) signaling in ROD

**Fig. 6:**
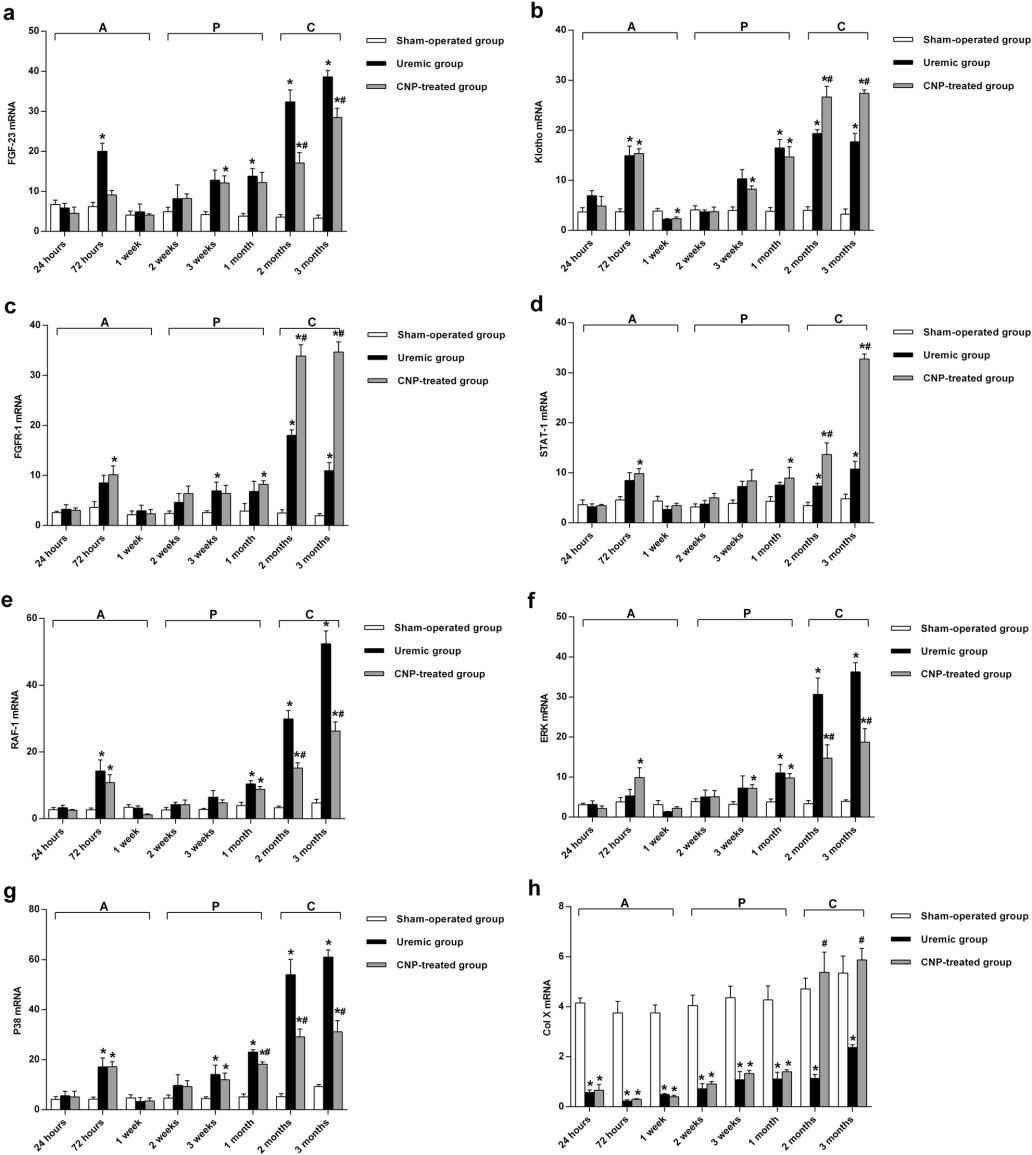
Real-time PCR analysis of bone FGF-23, Klotho, FGFR-1, STAT-1, RAF-1, ERK, P38, Col X, and GAPDH mRNA expression in sham-operated, uremic, and CNP-treated rats. Uremic group vs. sham-operated group: The uremic group had significant elevations in FGF-23, Klotho, RAF-1, and P38 mRNA expression in each stage (**a**, **b**, **e**, **g**); upregulations of FGFR-1 and ERK mRNA in the progressive stage and chronic stage (**c**, **f**); and overexpression of STAT-1 mRNA only in the chronic stage (**d**). CNP-treated group vs. uremic group: FGF-23 transcripts were significantly inhibited by exogenous CNP in the chronic stage (**a**); Klotho, FGFR-1, STAT-1, and Col X mRNA expression in the CNP-treated group was significantly higher in the chronic stage (**b**–**d**, **h**); CNP treatment initiated a dramatic suppression of P38, RAF-1, and ERK transcripts after 1, 2, and 2 months, respectively (**e**–**g**). CNP-treated group vs. sham-operated group: In comparison with the sham-operated group, Klotho, ERK, and P38 mRNA levels in the CNP-treated group significantly increased in every stage (**b**, **f**, **g**); FGF-23, FGFR-1, and RAF-1 mRNA were significantly elevated in the progressive stage and chronic stage (**a**, **c**, **e**); STAT-1 mRNA significantly increased in the chronic stage (**d**); Col X mRNA significantly decreased by 2 months postoperatively (**h**). ^*^P < 0.05, significantly different from the corresponding sham-operated group; ^#^*P* < 0.05, significantly different from the corresponding uremic group. A acute stage, P progressive stage, and C chronic stage

Bone expression levels of FGF-23, Klotho, FGFR-1, STAT-1, RAF-1, ERK, P38, and Col X mRNA at sacrifice in the uremic group and sham-operated group are shown in Fig. 6. The LSD test revealed that adriamycin significantly affected the expression of FGF-23/MAPK signaling and Col X mRNA (*t* ≥ 3.13, *P* < 0.01). The uremic group had significant elevations in FGF-23, Klotho, RAF-1, and P38 mRNA expression levels compared with the sham-operated group in each stage (all *P* < 0.05) (Fig. 6a, b, e and g), upregulated FGFR-1 and ERK mRNA levels in the progressive stage and chronic stage (both *P* < 0.05) (Fig. 6c, f), and overexpressed STAT-1 mRNA levels only in the chronic stage (*P* < 0.05) (Fig. 6d). In the uremic group, the highest expression levels of Klotho, FGFR-1, and P38 mRNA occurred at 2 months (3.83-fold, 6.24-fold and 9.34-fold, respectively), and the highest expression levels of FGF-23, STAT-1, RAF-1, and ERK mRNA were found at 3 months (10.63-fold, 1.23-fold, 10.06-fold, and 8.23-fold, respectively). In contrast, Col X mRNA in the uremic group was dramatically decreased and exhibited a largest downregulation (94% decrease) at 72 h (*P* < 0.05) (Fig. 6h).

**Fig. 7:**
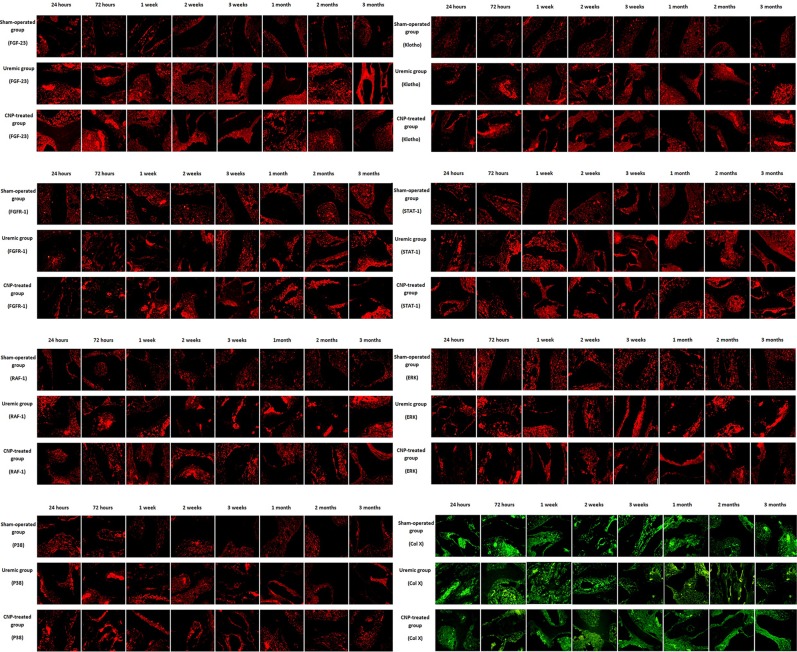
Immunofluorescence staining of FGF-23, Klotho, FGFR-1, STAT-1, RAF-1, ERK, P38, and Col X proteins in sham-operated, uremic, and CNP-treated rats. Uremic group vs. sham-operated group: There was no significant difference in positive immunostaining for FGF-23 between the two groups from 24 h to 1 month, whereas the divergence increased in a time-dependent manner and initially reached significance at 2 months. CNP-treated group vs. uremic group: Compared with the uremic group, the CNP-treated group showed higher positive immunostaining for Klotho, FGFR-1, STAT-1, and Col X proteins in the osteocytes in the chronic stage. In contrast, staining for RAF-1 and P38 protein was noticeably suppressed by CNP and displayed in a dot-linear manner after the acute stage

**Fig. 8:**
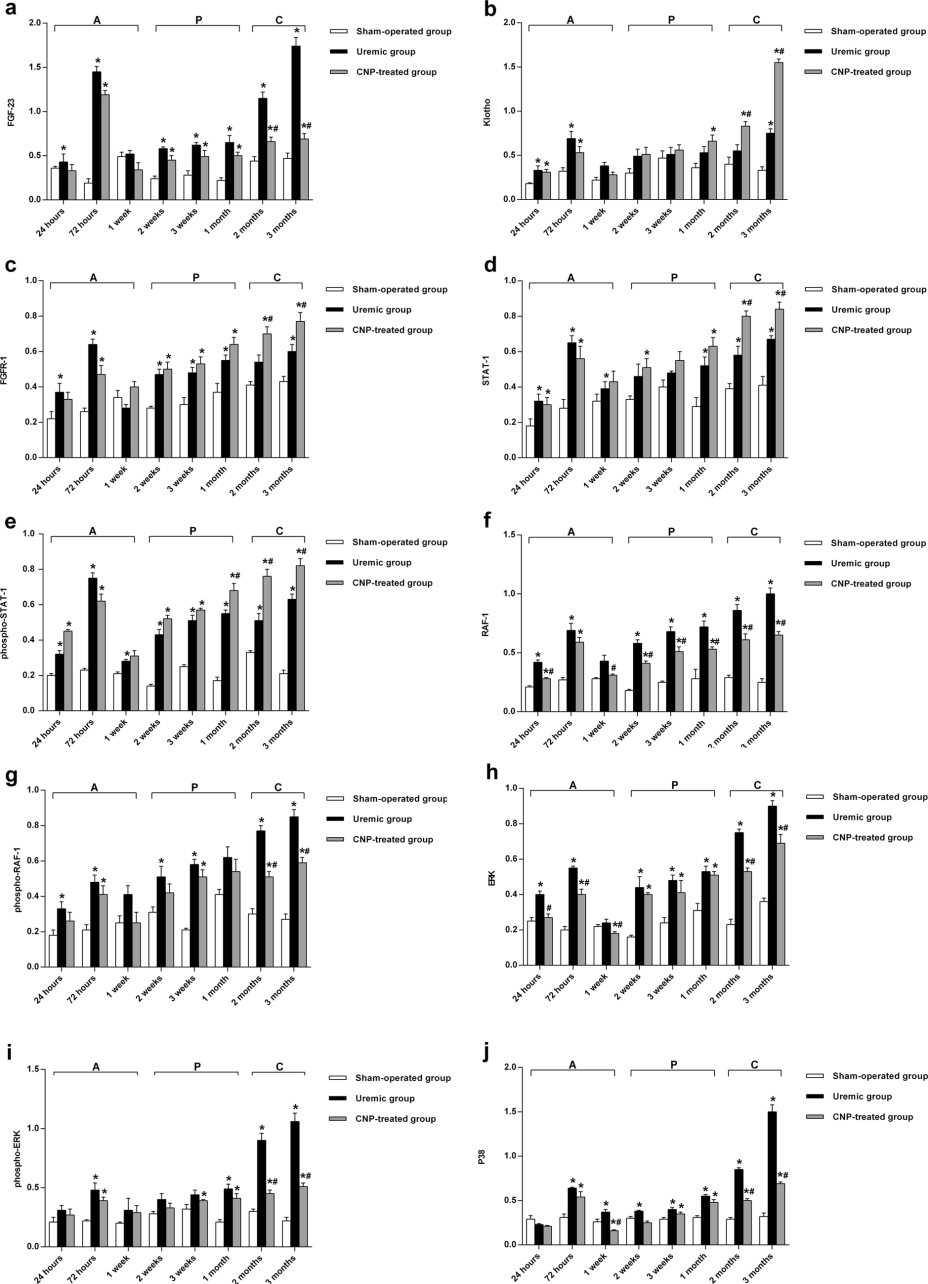

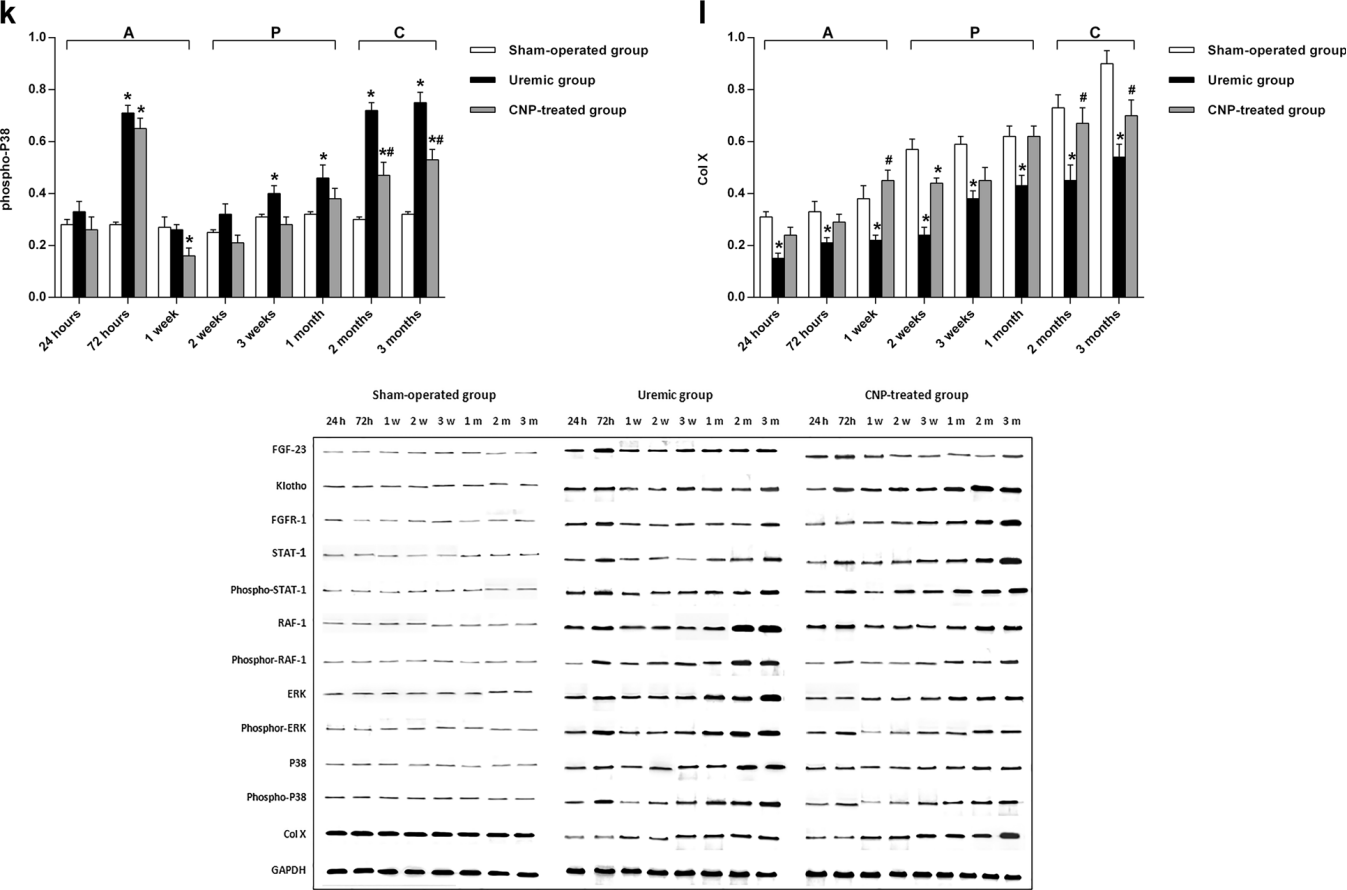
Western blotting to identify bone FGF-23, Klotho, FGFR-1, STAT-1, phosphor-STAT-1, RAF-1, phosphor-RAF-1, ERK, phosphor-ERK, P38, phosphor-P38, Col X, and GAPDH protein in sham-operated, uremic, and CNP-treated rats. Uremic group vs. sham-operated group: The uremic group experienced marked increases in FGF-23, FGFR-1, STAT-1, phospho-STAT-1, RAF-1, phospho-RAF-1, ERK, phospho-ERK, p38, and phospho-P38 protein in each stage (**a**, **c**–**k**); CNP-treated rats had higher Klotho protein levels in both the acute stage and chronic stage (**b**); the expression of Col X protein was significantly suppressed in the uremic group throughout the observational period (**l**). CNP-treated group vs. uremic group: CNP significantly reduced the expression of the FGF-23 protein in the chronic stage (**a**); CNP administration triggered significant elevations in Klotho, FGFR-1, and STAT-1 protein levels only in the chronic stage (**b**–**d**), upregulation of the phospho-STAT-1 protein level after the progressive stage (**e**), and overexpression of the Col X protein level in the acute stage and chronic stage (**l**), whereas RAF-1 and P38 protein levels were significantly suppressed by CNP throughout the observational period (**f**, **j**); The CNP-treated group showed significant reductions in phospho-RAF-1, phospho-ERK, and phospho-P38 protein levels in the chronic stage (**g**, **i**, **k**); CNP significantly downregulated the ERK protein level in both the acute stage and chronic stage (**h**). CNP-treated group vs. sham-operated group**:** In the CNP-treated group, the expression levels of FGF-23, Klotho, FGFR-1, STAT-1, phospho-STAT-1, RAF-1, phospho-RAF-1, phospho-ERK, ERK, and P38 were significantly higher than those in the sham-operated group in every stage (**a**–**j**); the phospho-P38 level was significantly elevated in the acute stage and chronic stage (**k**); the Col X level significantly decreased at 2 weeks postoperatively and was at a level similar to that in the sham-operated group after 3 weeks postoperatively (**l**). ^*^P < 0.05, significantly different from the corresponding sham-operated group; ^#^*P* < 0.05, significantly different from the corresponding uremic group. A acute stage, P progressive stage, and C chronic stage

To clarify the distributions of FGF-23, Klotho, FGFR-1, STAT-1, RAF-1, ERK, P38, and Col X protein between the uremic group and sham-operated group, immunofluorescence staining of the femur sections was performed (Fig. 7). Positive immunostaining for FGF-23, Klotho, FGFR-1, STAT-1, RAF-1, ERK, and P38 protein was detected in the osteocytes of the uremic rats in a clustered manner, whereas their distribution trends appeared scattered in the sham-operated group. Inversely, Col X protein was displayed in a puncture-like or dot-linear-like manner in the uremic group. Because changes in mRNA levels may not correspond to protein expression, western blotting was conducted for bone FGF-23, Klotho, FGFR-1, STAT-1, phospho-STAT-1, RAF-1, phospho-RAF-1, ERK, phospho-ERK, P38, phospho-P38, and Col X in this model (Fig. 8). The results from the LSD test indicated that FGF-23/MAPK signaling and Col X protein were significantly affected by adriamycin (*t* ≥ 7.0, *P* < 0.001). The uremic group experienced marked increases in FGF-23, FGFR-1, STAT-1, phospho-STAT-1, RAF-1, phospho-RAF-1, ERK, phospho-ERK, p38, and phospho-P38 protein levels compared with the sham-operated group in each stage (all *P* < 0.05) (Fig. 8a, c–k) and higher Klotho protein levels in both the acute stage and chronic stage (*P* < 0.05) (Fig. 8b). In the uremic group, the highest protein expression levels of FGF-23, FGFR-1, STAT-1, phospho-STAT-1 were detected at 72 h (6.63-fold, 1.46-fold, 1.32-fold, and 2.26-fold, respectively), the highest protein expression levels of ERK and phospho-P38 were found at 2 months (2.26-fold and 1.4-fold, respectively), and the highest protein expression levels of Klotho, RAF-1, phospho-RAF-1, phospho-ERK, and P38 were observed at 3 months (1.27-fold, 3-fold, 2.15-fold, and 3.68-fold, respectively). However, the protein expression level of Col X was significantly suppressed in the uremic group throughout the observational period and more specifically, exhibited the largest downregulation (57% decrease) at 2 weeks (*P* < 0.05) (Fig. 8l).

### Effect of CNP on FGF-23

The RT-PCR analysis of FGF-23 mRNA in the CNP-treated group and uremic group is shown in Fig. 6a. Two-way ANOVA showed that the expression of FGF-23 was significantly affected by main effects of treatment (*F*_treatment_ = 46.05, *P* < 0.001) and time (*F*_time_ = 18.29, *P* < 0.001) and an interaction between treatment and time (*F*_interaction_ = 7.01, *P* < 0.001). No significant difference in FGF-23 mRNA expression was observed between the two groups from 24 h to 1 month. When entering the chronic stage, expression of the FGF-23 transcript was significantly inhibited by exogenous CNP (a 47% decrease at 2 months and a 26% decrease at 3 months) (*P* < 0.05). The expression level of FGF-23 mRNA in the CNP-treated group was significantly higher than that of the sham-operated group in the progressive stage and chronic stage (*P* < 0.05).

The distribution change in the FGF-23 protein in the CNP-treated group and uremic group is illustrated in Fig. 7. Consistently, no significant difference in positive immunostaining for FGF-23 was found between the two groups from 24 h to 1 month, whereas the divergence was amplified in a time-dependent manner and initially reached significance after 2 months. The above characterization of FGF-23 expression was also confirmed using western blotting (Fig. 8a).

### Downstream signaling of FGF-23 after CNP administration

To determine the effect of CNP on the downstream signaling of FGF-23, we measured the bone abundance of Klotho, FGFR-1, STAT-1, RAF-1, ERK, P38, and Col X mRNA by RT-PCR (Fig. 6). It is evident that the main effects of treatment (*F*_treatment_ ≥ 40.54, *P* < 0.001) and time (*F*_time_ ≥ 18.88, *P* < 0.001) and the interaction between treatment and time (*F*_interaction_ ≥ 5.33, *P* < 0.001) significantly affected the downstream signaling of FGF-23 and Col X mRNA expression in the present study. Klotho, FGFR-1, STAT-1, and Col X mRNA levels in the CNP-treated group were significantly higher than those in the corresponding uremic group in the chronic stage (all *P* < 0.01) (Fig. 6b–d, h). The highest expression level of Col X mRNA was observed at 2 months (3.75-fold), and the highest expression level of Klotho, FGFR-1, and STAT-1 was found at 3 months (0.57-fold, 2.17-fold, and 2.05-fold, respectively). CNP treatment initiated a dramatic suppression of P38, RAF-1, and ERK transcripts at 1, 2, and 2 months, respectively (all *P* < 0.05) (Fig. 6e–g); more specifically, the largest downregulation among these transcripts occurred at 3 months (a 49% decrease in P38), 3 months (a 50% decrease in RAF-1), and 2 months (a 42% decrease in ERK). In comparison with the sham-operated group, the CNP-treated group showed a significant increase in Klotho, ERK, and P38 mRNA levels in the in every stage, in FGFR-1 and RAF-1 mRNA levels in the progressive stage and chronic stage, and in the STAT-1 mRNA level in the chronic stage, whereas the Col X mRNA level significantly decreased within the first 2 months postoperatively (all *P* < 0.05) (Fig. 6).

The distributional characterizations of Klotho, FGFR-1, STAT-1, RAF-1, ERK, P38, and Col X proteins in the CNP-treated group and uremic group were determined by immunofluorescence staining (Fig. 7). Compared with the uremic group, stronger positive immunostaining for Klotho, FGFR-1, STAT-1, and Col X proteins was located in the osteocytes of the CNP-treated group in the chronic stage. In contrast, staining for RAF-1 and P38 proteins was clearly suppressed by CNP and displayed in a dot-linear manner after the acute stage.

The FGF-23 downstream signaling proteins expressed in femurs were analyzed by western blotting (Fig. 8). A two-way ANOVA revealed that the downstream expression of FGF-23 and Col X signaling proteins were significantly affected by the main effects of treatment (*F*_treatment_ ≥ 44.42, *P* < 0.001) and time (*F*_time_ ≥ 11.44, *P* < 0.001) and the interaction between treatment and time (*F*_interaction_ ≥ 2.91, *P* < 0.001). CNP administration triggered significant elevations in Klotho, FGFR-1, and STAT-1 protein levels only in the chronic stage (Fig. 8b–d), upregulation of the phospho-STAT-1 protein after the progressive stage (Fig. 8e), and overexpression of the Col X protein in the acute stage and chronic stage (all *P* < 0.05) (Fig. 8l). The highest protein expression levels of FGFR-1, STAT-1, and phospho-RAF-1 were observed at 2 months (0.3-fold, 0.38-fold, and 0.49-fold, respectively) and the highest protein expression levels for Klotho and Col X were found at 3 months (1.07-fold) and 1 week (1.05-fold), respectively. Notably, RAF-1 and P38 protein expression was significantly suppressed by CNP throughout the observational period (both *P* < 0.05) (Fig. 8f, j). The CNP-treated group showed significant decreases in phospho-RAF-1, phospho-ERK, and phospho-P38 protein levels in the chronic stage (Fig. 8g, i, k) and downregulated the ERK protein level in both the acute stage and chronic stage (all *P* < 0.05) (Fig. 8h). The largest downregulation of RAF-1, phospho-ERK, and P38 protein levels was detected at 3 months (35% decrease, 52% decrease, and 64% decrease, respectively), and the largest downregulation of phospho-RAF-1, ERK, and phospho-P38 protein levels was observed at 2 months (34% decrease, 29% decrease, and 35% decrease, respectively). In the CNP-treated group, the expression levels of Klotho, FGFR-1, STAT-1, phospho-STAT-1, RAF-1, phospho-RAF-1, phospho-ERK, ERK, and P38 were significantly higher than those in the sham-operated group in every stage, and phospho-P38 was significantly elevated in the acute stage and chronic stage; however, Col X significantly decreased at 2 weeks postoperatively and had a level similar to that in the sham-operated group at 3 weeks postoperatively (all *P* < 0.05) (Fig. 8).

## Discussion

In the present study, we established a ROD model to determine whether CNP is capable of retarding ROD progression in vivo. The uremic rats manifested proteinuria, hypoalbuminemia, renal dysfunction, and renal pathological changes, consistent with previous studies^25,26^. On the other hand, adriamycin induced calcium phosphate metabolic disorders, hypovitaminosis D, and secondary hyperparathyroidism. The 2017 Kidney Disease: Improving Global Outcomes (KDIGO) Guideline suggests that 25-(OH) D levels should be measured and monitored during the therapeutic period in CKD patients^27^. Marckmann et al. conducted an 8-week randomized, double-blind parallel intervention study of 52 CKD patients, and revealed that serum levels of 25-(OH) D were almost 1000 times higher than 1, 25-dihydroxyvitamin D; in addition, serum 25-(OH) D was positively correlated with serum 1, 25-dihydroxyvitamin D, calcium, and FGF-23 levels, whereas it was inversely correlated with PTH levels. However, no association of 1, 25-dihydroxyvitamin D with these above biomarkers was found, which may be attributed to the lower sensitivity and shorter half-life of 1, 25-dihydroxyvitamin D in circulation^28^. On this background, 25-(OH) D was assessed in the present study rather than the active form of vitamin D. Elevated PTH levels increased calcium efflux from bone and renal calcium resorption, while a decrease in 1, 25-dihydroxyvitamin D impaired internal calcium absorption. The resistance of bone to PTH has been observed in CKD progression^29^, and therefore, increased levels of PTH only partially counteract hypocalcemia induced by dramatic reductions in Vitamin D^30^. Moreover, decreased phosphate levels throughout the observational period could be caused by elevated FGF-23 through increasing phosphate excretion, reducing phosphate resorption and increasing PTH secretion, further enhancing phosphate excretion^31^. The observational period of our study may have been too short to cover every stage of the disease. Therefore, low phosphate occurred only from the disease initiation to 3 months postoperatively. Na/Pi transporters and 1α-hydroxylase are well known to be responsible for the regulation of phosphate and 1, 25-dihydroxyvitamin D, respectively. Hasegawa and Sugiura established a CKD model through injections of anti-glomerular basement membrane antiserum plus a high phosphate diet and found that the expression of Na/Pi transporters and 1α-hydroxylase exhibited more than a 50% reduction in the kidney after the 7 weeks, which could be reversed by FGF-23 antibody^32,33^. The uremic rats displayed lower levels of osteoblastic and osteolytic proteins, which was consistent with previous studies showing that tAP, bAP, DPD, and P1NP were decreased in CKD^34,35^. In addition, bone pathological progression associated with adriamycin was attributed to low-turnover ROD. In 1980, Van Vleet and Ferrans first studied chronic intoxication with adriamycin (2.4 mg/kg/week) in 50 weanling rabbits for up to 17 weeks, showing that prominent renal damage was selective for the inner cortex and that other lesions included skeletal muscle degeneration and osteodystrophy-associated fractures^36^. Accordingly, 2 decades later, Ishii et al. established a uremic model in Sprague-Dawley rats by a single peritoneal injection of 5 mg/kg adriamycin and found that GFR and serum 25-(OH) D were reduced by 52% and 77%, respectively, whereas PTH experienced a 2.5-fold increase after 14 weeks; moreover, the adriamycin-injected rats could also develop low-turnover ROD resembling osteomalacia, as evidenced by a 4.3-fold increase in osteoid volume, a 73% reduction in BFR, a 56% decrease in adjusted apposition rate, and a five-fold increase in mineralization lag time^34^. Another study conducted by Graciolli et al. indicated that CKD patients also exhibited low-turnover ROD, characterized by higher osteoid volume and mineralization defect despite the continuous increase in PTH levels^37^. On the basis of this evidence, we identified our model as low-turnover ROD although secondary hyperparathyroidism exists. The 2017 KDIGO Guideline suggests that serum calcium levels are positively associated with increases in bone mineral density in CKD children and that this association is significantly more pronounced with greater linear growth velocity^27^. In addition, a univariate linear regression demonstrated that both calcium and phosphate showed a linear association with bone formation rate; more specifically, the change in calcium played a more relevant role in bone pathology^38^. Although PTH and vitamin D have been proved to be involved in the regulation of mineral metabolism, calcitonin may also provide an insight into ROD pathogenesis. Calcitonin can participate in the phase control and period entrainment of spontaneous periodic bone calcium efflux via the circadian profile of serum calcitonin levels^39^.

FGF-23, a bone-derived endocrine regulator of phosphate homeostasis, downregulates bone mineralization and promotes osteolysis. Teerapornpuntakit et al. incubated rat osteoblast-like UMR-106 cells with FGF-23 for 48 h and found that cell proliferation was significantly suppressed and that osteoblast-derived osteoclastogenic factors were upregulated by FGF-23 in a dose-dependent manner^40^. Generally, CNP is considered an endothelium-derived peptide and positively regulates bone formation. Lenz et al. cultured CRL-11372 human osteoblast cells with different concentrations of CNP (10, 100 pM, 1, and 10 nM) for 7 days and revealed that the pro-osteoblastic effect of CNP also showed a dose-specific response; 10 nM CNP exerted the highest acceleration of osteoblast proliferation (~30%)^41^. To the best of our knowledge, elevated FGF-23, together with decreased CNP, is often observed in CKD. Numerous clinical trials have demonstrated that FGF-23 levels rise in parallel with declining renal function^42,43^. In a previous study, we observed that both urinary excretion and renal expression of CNP were markedly upregulated in the early stages of CKD, whereas they progressively declined with disease progression^22^. Since the effects of CNP and FGF-23 on bone formation appear to oppose each other, it is reasonable to propose that their interaction is involved in ROD. However, there are three additional mysteries remaining to be solved.

The first is whether FGF-23 is involved in the progression of ROD. In the present study, bone expression of FGF-23 was significantly elevated in ROD and characterized by a bimodal pattern with the progression of the disease; the first peak was at 72 h, and the second was at 3 months. Similarly, Graciolli et al. enrolled 148 CKD patients to probe the underlying mechanisms of ROD and found that the elevated FGF-23 level was correlated inversely with bone formation rate, positively correlated with a longer mineralization lag time^37^. In a mouse model of ROD established by two-step electrocautery, the elevation in FGF-23 level could reflect the degree of mineral disorders and secondary hyperparathyroidism^44^. In this circumstance, FGF-23 may play a crucial role in ROD pathogenesis. However, whether increased levels of FGF-23 in bone directly results in the initiation of ROD is still unclear to date. FGF-23 binds to four FGFRs in the presence of Klotho, and among the four receptors, FGFR-1 has the highest affinity for FGF-23^45^. Accordingly, the present study assessed the bone expression of Klotho and FGFR-1 in ROD and showed that both of them were significantly upregulated. However, in previous studies on ROD, the expression of Klotho and FGFR-1 appeared to be suppressed in vessels and parathyroid glands, whereas it was enhanced in circulation^44,46^. It seems that Klotho and FGFR-1 are paradoxically expressed between bone and the other tissues, and we speculate that the increased circulating levels of Klotho and FGFR-1 may be attributed, in part, to the upregulated secretion from bone. An in vitro study elucidated that exogenous FGF-23/Klotho increased FGFR-1 expression in osteoblast-like cells^47^. MAPK signaling acts as a common pathway for the bone metabolism regulated by FGF-23 and includes four key elements (STAT-1, ERK, RAF-1, and P38)^48^. Our study demonstrated that STAT-1, ERK, RAF-1, P38, and their phosphorylated forms were significantly increased in bone tissue. As far as we know, limited studies have focused on the above four elements of MAPK signaling involved in the ROD pathogenesis to date. A pioneer study published in *Kidney International* suggested that the Wnt pathway of the rat osteosarcoma cell line UMR106-01 could be significantly suppressed by exposure to uremic serum. To clarify which factors may be responsible for Wnt inactivation, Carrillo-Lo´pez et al. further cultured UMR106-01 cells with FGF-23/Klotho and found a prominent elevation in the phospho-ERK/ERK ratio; on the contrary, when UMR106-01 cells were exposed to FGF-23/Klotho in the presence of a MAPK inhibitor, the upregulation of the phospho-ERK/ERK ratio was abolished^49^. The above findings provide a novel mechanism of the adverse impact of FGF-23 on bone metabolism in CKD through a soluble Klotho/MAPK process.

Second, does CNP act as a potential therapy for ROD through antagonizing FGF-23? In the present study, we probed the therapeutic effect of CNP on both uremia and ROD progression and revealed that renal function, calcium phosphate metabolic disorders, hypovitaminosis D, secondary hyperparathyroidism, and decreased bone turnover markers were restored by CNP to some extent; directly, CNP injection could also improve bone pathological changes through increasing osteoblasts, osteoclasts, osteoid volume, and trabecular thickness in the uremic rats. Interestingly, UCr was increased by CNP treatment throughout the whole observational period based on our findings. In an isolated perfused rat kidney model, Wei and Alves observed that CNP offset the vasoconstriction effect induced by phenylephrine and improved GFR significantly^50,51^, which may lead to the increase in UCr in the present study. Moreover, the present study indicated that CNP significantly downregulated the bone expression of FGF-23 only in the chronic stage. A good correlation between serum levels and bone expression of FGF-23 was verified in a previous study^37^. Therefore, we speculate that serum FGF-23 may have a consistent partial rescue with FGF-23 in bone after CNP infusion. Although bone integrity is impacted by the regulation of phosphate and 1, 25-dihydroxyvitamin D, it is still difficult to identify whether the activity of FGF-23 could be antagonized by CNP directly in the bone based on our present findings. Subsequent studies on osteoblasts cultured with CNP/NPR-B inhibitor have been undertaken in our laboratory to confirm the present animal research results (data not shown).

Third, how does CNP suppress FGF-23/MAPK signaling in ROD? To further elucidate whether the therapeutic effect of CNP on ROD is mediated by inactivating FGF-23, we assessed four key elements of MAPK signaling (STAT-1, ERK, RAF-1, and P38); among them, RAF-1 was proved to be a specific node contributing to the interaction of CNP and FGF-23. Within FGF-23/MAPK signaling, the FGFR-1, Klotho, and alternative (STAT-1/phospho-STAT-1) elements were upregulated by CNP, whereas RAF-1/phospho-RAF-1 and its downstream (ERK/phospho-ERK and P38/phospho-P38) elements were paradoxically underexpressed in bone tissue. Currently, a few studies have documented the antagonistic mechanism of CNP against the FGF family in vitro. Krejci et al. treated rat chondrosarcoma cells with CNP and FGF-2 for 30 min and found that CNP abolished FGF-2-mediated ERK activation at the level of RAF-1^52^. Hence, the evidence from our laboratory and Krejci’s panel supports that CNP antagonizes FGF-23 through modulating not only MAPK expression but also its activation. In another study, the inhibitory effects of chondrocyte proliferation were reversed from 24.7% to 88% in STAT-1 knockout mice after FGF stimulation compared with those in wild-type mice, which suggests that STAT-1 function is also required for FGF-mediated growth inhibition^53^.

In summary, our study revealed, for the first time, that FGF-23 may be involved in ROD pathogenesis and that exogenous CNP could exert a therapeutic effect on ROD through inhibition of FGF-23/MAPK signaling at the RAF-1 level. Information on the therapeutic effect and underlying mechanism of CNP on FGF-23/MAPK is still scarce to date. Currently, an in vitro study on osteoblasts cultured with CNP and FGF-23 inhibitor is being conducted in our laboratory, and the preliminary data are being arranged and analyzed.

## Supplementary information

Supplementary Table
