# Supplementary material for: C-type natriuretic peptide attenuates renal osteodystrophy through inhibition of FGF-23/MAPK signaling
Source: Exp Mol Med. 2019 Jul 1;51(7):70. doi: 10.1038/s12276-019-0265-8 (PMC6802631; doi:10.1038/s12276-019-0265-8)
Supplement: Supplementary file 1 — Supplementary Table [file 12276_2019_265_MOESM1_ESM.docx]

**Supplemental table Renal function indices between the uremic group and sham-operated group**

| **Stage** | **Group** | **Alb (g/L)** | **BUN (mmol/L)** | **SCr (umol/L)** | **UA (umol/L)** | **UCr (umol/L)** | **Upr (mg)** |
| --- | --- | --- | --- | --- | --- | --- | --- |
| **Acute stage** | Sham-operated group | 34.65±2.21 | 6.96±1.26 | 18.22±6.75 | 62.40±5.01 | 31.98±6.27 | 3.05±0.77 |
|  | Uremic group | 32.12±2.77**↓** | 12.04±4.69**↑** | 17.57±3.94 | 67.35±7.42**↑** | 39.83±40.4 | 3.93±1.70 |
| **Progressive**  **stage** | Sham-operated group | 34.22±1.83 | 6.74±1.44 | 20.21±5.19 | 60.78±2.46 | 31.18±5.47 | 3.26±0.75 |
|  | Uremic group | 30.02±3.72**↓** | 9.42±1.27**↑** | 24.78±2.67**↑** | 74.99±21.05**↑** | 135.02±137.14**↑** | 10.73±11.03**↑** |
| **Chronic stage** | Sham-operated group | 34.83±2.26 | 6.40±1.21 | 19.13±7.95 | 60.48±4.41 | 31.51±7.42 | 3.27±3.15 |
|  | Uremic group | 31.76±4.06**↓** | 10.14±3.01**↑** | 33.63±3.95**↑** | 83.00±7.16**↑** | 1363.47±622.33**↑** | 8.53±1.01**↑** |
